# Signaling Mechanisms of Endogenous Angiogenesis Inhibitors Derived from Type IV Collagen

**DOI:** 10.4137/grsb.s345

**Published:** 2007-10-14

**Authors:** Akulapalli Sudhakar, Chandra S. Boosani

**Affiliations:** 1 Cell Signaling and Tumor Angiogenesis Laboratory, Department of Genetics, Boys Town National Research Hospital, Omaha, NE 68132; 2 Department of Biomedical Sciences, Creighton University, School of Medicine, Omaha, NE, 68178; 3 Department of Biochemistry and Molecular Biology, University of Nebraska Medical Center, Omaha, NE, 68198

## Abstract

Vascular basement membrane (VBM) derived molecules are regulators of certain biological activities such as cell growth, differentiation and angiogenesis. Angiogenesis is regulated by a systematic controlled balance between VBM derived antiangiogenic factors and proangiogenic growth factors. In the normal physiological state, equilibrium is maintained between the antiangiogenic and proangiogenic factors. The antiangiogenic factors (molecules), which are generated by the proteolytic cleavage of the VBM, include; α1 chain non-collagenous (NC1) domain of type XVIII collagen (endostatin) and the NC1 domains from the alpha chains of Type IV collagen considered as endogenous angiogenesis inhibitors. These collagen derived NC1 domains have a pivotal role in the regulation of tumor angiogenesis, thus making them attractive alternate candidates for cancer therapies. In this review we illustrate a comprehensive overview of the knowledge gained from the signaling mechanisms of Type IV collagen derived endogenous inhibitors in angiogenesis.

## Introduction

Angiogenesis, the sprouting of capillaries from pre-existing blood vessels, or by splitting of blood vessels is among the key events in destructive pathological processes such as tumor growth, metastasis, arthritis, age related macular degeneration etc., as well as in physiological processes such as development, organ growth, reproduction and wound healing (Folkman, 1995a). Folkman’s group first reported a hypothesis that tumor growth is dependent on neovascularization or angiogenesis (Folkman, 1995a; Folkman, 1995b). The growth of tumors is strictly dependent on the neovascularization, and the inhibition of vascular supply to tumors can suppress tumor growth (Folkman, 1971; Hanahan and Folkman, 1996). Solid tumors cannot grow beyond 2 to 3 mm in diameter without recruitment of their own blood supply, thus tumor angiogenesis results from a balance between endogenous activators [vascular endothelial growth factor (VEGF), fibroblast growth factor (FGF), and platelet-derived growth factor (PDGF) etc.] and inhibitors [various antiangiogenic peptides generated from VBM or extracellular matrix (ECM) degradation by proteases] (Folkman, 1995a; Kieran et al. 2003; Folkman, 2003).

Endogenous angiogenesis inhibitors from ECM includes a large multifunctional ECM glycoproteins such as thrombospondin (Good et al. 1990), Endorepellin, a COOH terminal end of perlecan, (or perlecan domain V) (Yurchenco and O’Rear, 1994), Anastellin, a fibronectin fragment, Fibulins (COOH terminal fragments corresponding to fibulin 1D and the domain 111 of fibulin 5) (Yi and Ruoslahti, 2001; Albig and Schiemann, 2004). Endostatin, a 20 kDa fragment derived from the COOH-terminal non-collagenous domain of α1 chain of type XVIII collagen (O’Reilly et al. 1997) and Type IV collagen derived α1 chain non-collagenous α1(IV)NC1, α2(IV)NC1, α3(IV)NC1 and α6(IV)NC1 domains (Petitclerc et al. 2000).

Non-ECM derived endogenous angiogenesis inhibitors includes angiostatin, a 38 to 45 kDa peptide from plasminogen, that contain homologous triple-disulfide bridged kringle domains, 1 to 4 or 1 to 3 (Patterson and Sang, 1997; Cornelius et al. 1998). Circulating clotting factors in the blood are also known to play an important role in angiogenesis. These factors include Antithrombin III, a latent form of intact antithrombin (O’Reilly et al. 1999), Prothrombin kringle-2, is derived from cleavage of the COOH-terminal loop of antithrombin and the cleaved conformational changed molecule showing antiangiogenic and antitumorogenic activity (Lee et al. 1998). Tissue inhibitors of matrix metalloproteinases-2 (TIMP-2) suppress MMP activity and ECM turnover (Brew et al. 2000; Jiang et al. 2002), 2-Methoxyestradiol (2-ME) an endogenous estradiol metabolite (Mabjeesh et al. 2003), Vasostatin, a NH2-terminal domain of human Calreticulin inclusive of 1,180 amino acids (Pike et al. 1998; Pike et al. 1999), soluble Fms-like tyrosine kinase 1 (sFlt-1) or VEGFR1 (Kendall and Thomas, 1993), Troponin I (Tn I) derived from cartilage (Moses et al. 1999), Pigment epithelium-derived factor (PEDF), a non-inhibitory member of the serpin superfamily (Volpert et al. 2002), Interferon α/β (INFα/β) (Lingen et al. 1998; Dinney et al. 1998), Chondromodulin-I, a 25 kDa cartilage specific Non-Collagenous-1 matrix protein (Kusafuka et al. 2002), PEX, a non-catalytic COOH terminal hemopexin-like domain of MMP-2 (Brooks et al. 1998), Prolactin fragment, 16 kDa and 8 kDa fragments generated from 23 kDa intact prolactin (Ferrara et al. 1991), Interleukins (a family of leukocyte-derived proteins) (Strieter et al. 1995b; Strieter et al. 1995a) and platelet factor-4 (release from platelet α-granules during platelet aggregation) (Maione et al. 1990) etc.

This review will highlight some of the important features of Type IV collagen-derived angiogenic inhibitor molecules and address their integrin mediated signaling mechanisms in the regulation of abnormal neovascularization in tumors, that would explain how these endogenous angiogenesis inhibitors regulate angiogenic balance in the tumor bed.

## Type IV Collagen Derived Angiogenesis Inhibitors

Type IV collagen is the most abundant constituent of the basement membrane (BM) that forms a network like structure in the extracellular matrix. Type IV collagen providing a scaffold in the BM with other macromolecules, such as laminins, heparan sulfate proteoglycans, fibronectin, entactin and regulates the interaction with adhering cells (Timpl et al. 1981; Kuhn et al. 1981; Timpl, 1996). Type IV collagen is found normally only in the BM, but during pathogenesis, it is associated with tumor fibrosis and accumulates in the tumor inter-stitium (Timpl et al. 1981; Kuhn et al. 1981). Type IV collagen is composed of six (α1 to α6) distinct gene products and their genomic localization shows a pair-wise head-to-head arrangements with a bi-directional promoter, that were mapped onto three different chromosomes (Hudson et al. 1993; Hudson et al. 1994; Kuhn, 1995). α1 and α2 chains are most abundant forms of Type IV collagen found in most basement membranes (BM) (Hudson et al. 2003). Where as α3–α6 chains are found in kidney a specialized glomerular basement membrane with specific functional properties (Hudson et al. 2003).

The [α1(IV)]_2_α2(IV) trimers contain a triple helical domain with binding sites for α1β1 and α2β1 integrins (Vandenberg et al. 1991). Initially in 1986, cells binding to Type IV collagen and its inhibition with Type IV collagen peptides has been demonstrated (Aumailley and Timpl, 1986; Tsilibary et al. 1990; Chelberg et al. 1990). Tsilibary in 1990, first reported a peptide that was derived from non-collagenous domain (NC1) of the α1(IV) chain could promote adhesion of bovine aortic endothelial cells (Tsilibary et al. 1990). The functional α1 and α2 Type IV collagen chains isolated from the Engelbreth Holm Swarm Sarcoma tumors inhibited capillary endothelial cell proliferation (Ries et al. 1995; Madri, 1997).

The new functions for α2, α3 and α6 NC1 domains of type IV collagen and their integrin ligands inhibiting angiogenesis and tumor growth in vivo reported in 2000 (Petitclerc et al. 2000). Later several laboratories worked on these molecules and further supported antiangiogenic and antitumorogenic activities of these NC1 domains (Kamphaus et al. 2000; Maeshima et al. 2000; Pasco et al. 2000; Colorado et al. 2000; Marneros and Olsen, 2001; Maeshima et al. 2002; Sudhakar et al. 2003; Hamano et al. 2003; Sudhakar et al. 2005; Roth et al. 2005; Magnon et al. 2005; Borza et al. 2006; Boosani and Sudhakar, 2006; Boosani et al. 2007; Magnon et al. 2007). The molecular signaling mechanisms for regulation of angiogenesis by α1, α2, α3 and α6 NC1 domains of Type IV collagen are updated in this review. Understanding the mechanism(s) of action of such molecules would aid in unraveling their therapeutic applications.

### α1(IV)NC1 or arresten

α1(IV)NC1 is one of the recently identified endogenous inhibitors of angiogenesis. It is a 26-kDa molecule derived from the NC1 domain of the α1 chain of Type IV collagen by proteases (Colorado et al. 2000; Sudhakar et al. 2005; Boosani et al. 2006). The extensive studies from my laboratory and others suggest that α1(IV)NC1 functions via α1β1 integrin and blocks the binding of α1β1 integrin to the Type IV collagen (Colorado et al. 2000; Sudhakar et al. 2005). Integrin α1β1 is a collagen binding receptor that also binds to other basement membrane components such as laminin (Zutter and Santoro, 1990; Keely et al. 1995). Both α1 and β1 integrins are involved in angiogenesis (Senger et al. 2002). Using the neutralizing antibodies for α1 integrin, angiogenesis associated with tumor growth could be suppressed. Blocking of α1β1 integrin interactions with ECM inhibits angiogenesis, which indicates that the integrins α1β1 acts as proangiogenic receptors (Senger et al. 2002). Among the integrin receptors for collagen, α1β1 integrin activates the Ras/Shc mitogen activated protein kinase (MAPK) pathway promoting cell proliferation (Senger et al. 2002). We demonstrated that α1(IV)NC1 binds to α1β1 integrin in a collagen type IV dependent manner and mediates all of its antiangiogenic functions through this integrin and inhibits angiogenesis by inhibiting endothelial cell proliferation, migration and tube formation (Sudhakar et al. 2005; Boosani et al. 2006). α1(IV)NC1 might also function via binding to heparan sulfate proteoglycans. Previously heparan sulfate proteoglycan was reported to bind to α1(IV)NC1 domain (Colorado et al. 2000). Significant halt in pathological angiogenesis and tumor growth was reported in α1 integrin knockout mice (Pozzi et al. 2000; Sudhakar et al. 2005). Whereas, α1(IV)NC1 had no effect in α1 integrin knockout mouse lung endothelial cells (Sudhakar et al. 2005). On the contrary, it significantly inhibited proliferation of wild type mouse lung endothelial cells. Thus confirms the significance of integrin mediated signaling of α1(IV)NC1 (Sudhakar et al. 2005).

In endothelial cells, ligand upon binding to integrins induces FAK phosphorylation, which serves as a platform for different downstream signals (Hynes, 2002; Kim et al. 2002; Sudhakar et al. 2003). Classical integrin ligand interactions are known to initiate intracellular signaling pathways, however some of such signaling events are reported to be inhibited by α1(IV)NC1 by binding to α1β1 integrin (Sudhakar et al. 2005). α1(IV)NC1 inhibits phosphorylation of FAK when mouse lung endothelial cells (MLEC) are plated on collagen type IV matrix (Sudhakar et al. 2005). Similar inhibition of FAK phosphorylation was not observed with α1(IV)NC1 treatment in α1 integrin knockout MLEC cells (Sudhakar et al. 2005). Downstream to FAK, protein kinase B (Akt/PKB) plays an important role in endothelial cell survival signaling (Shiojima and Walsh, 2002; Sudhakar et al. 2003; Sudhakar et al. 2005). α1(IV)NC1 does not inhibit Akt or phosphatidyl-3-kinase (PI3 kinase) phosphorylation suggesting that α1(IV) NC1 regulates migration of endothelial cells in an Akt-independent manner (Sudhakar et al. 2005).

**Figure 1:**
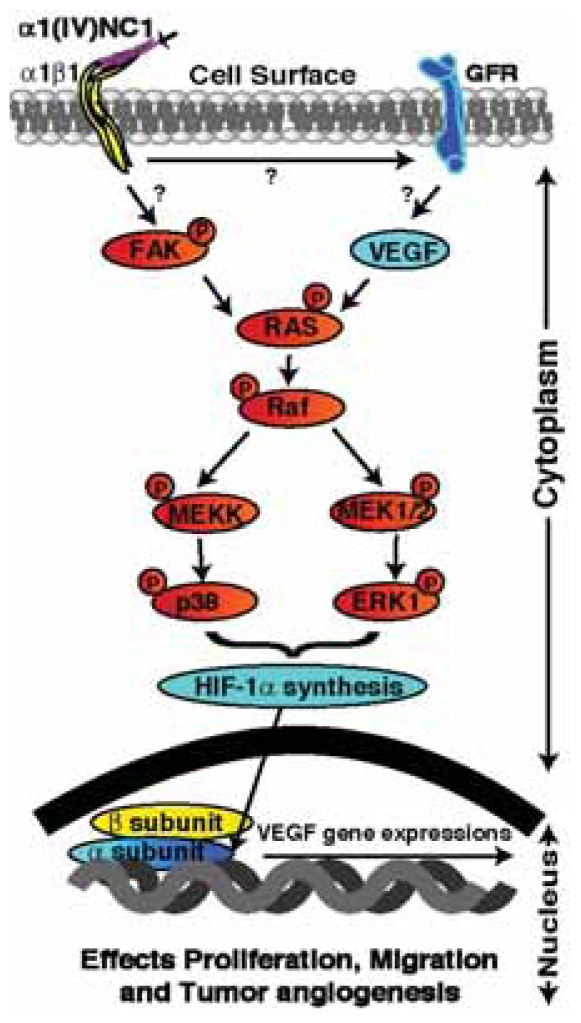
Schematic illustration of signaling pathway mediated by α1(IV)NC1. α1(IV)NC1 binds to α1β1 integrin and cross talk with growth factor receptors, and inhibit phosphorylation of FAK. Inhibition of FAK activation leads to inhibition of Raf/MEK/ERK1/2/p38 MAP kinase pathways that leads to inhibition of HIF-1α and VEGF expression which in turn results in inhibition of endothelial cell migration, proliferation and tube formation in proliferating endothelial cells.

Interestingly hypoxia induced factor alpha (HIF-1α) expression was inhibited by treatment of α1(IV)NC1 in hypoxic (lack of oxygen) endothelial cells (Sudhakar et al. 2005). HIF-1α is an oxygen-dependent transcriptional activator, which plays crucial roles in the tumor angiogenesis (Semenza, 2003; Lee et al. 2004). HIF-1α regulates cellular responses to physiological and pathological hypoxia, and studies demonstrate that HIF-1α is a potential target for tumor angiogenesis (Wu et al. 2003; Unruh et al. 2003). HIF-1α transcriptionally regulates VEGF expression in hypoxic cells and promotes angiogenesis in solid tumors (Kung et al. 2000; Miller et al. 1994; Carmeliet et al. 1998; Sudhakar et al. 2005). These findings suggest that HIF-1α is a prime target for anticancer therapies. Our recently published findings demonstrate that α1(IV)NC1 binds to α1β1 integrin on endothelial cells and inhibits MAPK signaling, which results in inhibition of HIF-1α expression (Fig. 1) (Sudhakar et al. 2005). Wild type tumor bearing mice when treated with α1(IV)NC1, decreased circulating VEGFR2 positive endothelial cells, and such observations were not seen in MLECs of integrin α1 knockout mice. Measuring the number of circulating endothelial cells is being evaluated as pharmacodynamic marker (Hurwitz et al. 2004). These studies provide a rationale for the use of α1(IV)NC1 as an inhibitor of HIF-1α and VEGF in hypoxic endothelial cells (Sudhakar et al. 2005). This hypoxic inhibitory activity might be exploited for antiangiogenic therapy in the treatment of cancer, but more pre-clinical laboratory studies are needed.

### α2(IV)NC1 or canstatin

Proteolytic degradation of type IV collagen liberates a 24-kDa peptide from α2 chain, called α2(IV)NC1, this peptide was reported to inhibit tumor associated angiogenesis (Petitclerc et al. 2000). The exact mechanisms by which this NC1 domain of TypeIV collagen inhibits tumor angiogenesis is not completely understood. α2(IV)NC1 binds to the endothelial and tumor cell surface in an αVβ3 and αVβ5 integrin dependent manner (Panka and Mier, 2003; Roth et al. 2005; Magnon et al. 2005; Magnon et al. 2007). α2(IV)NC1 competes with Type IV collagen of ECM for cell surface integrin binding and reverses the proliferative and migratory effects induced by cell-ECM interactions (Kamphaus et al. 2000). Thus, αVβ3 and αVβ5 integrins appear to mediate the antiangiogenic and antitumorgenic properties of α2(IV)NC1 (Magnon et al. 2005). In addition, researchers also determined that α2(IV)NC1 binds to αVβ3 and αVβ5 integrins and induce apoptosis in endothelial and certain tumor cells (Magnon et al. 2005). α2(IV)NC1 inhibits the growth of many tumors in human xenograft mouse models, histological studies revealed decreased CD31 positive vasculature (Petitclerc et al. 2000; Kamphaus et al. 2000; Roth et al. 2005; Magnon et al. 2005; Magnon et al. 2007).

α2(IV)NC1 strongly inhibits the migration and proliferation of endothelial cells. Moreover, these events are mediated by an upstream event involving α2(IV)NC1 binding to αVβ3 and αVβ5 integrins. Recent findings have shown that α2(IV)NC1 inhibits the phosphorylation of Akt, FAK, mammalian target of rapamycin (mTOR), eukaryotic initiation factor 4E binding protein-1 (4E-BP1), and ribosomal S6 kinase in cells (Panka and Mier, 2003). Collectively, the available research information suggests that, α2(IV)NC1 binds to αVβ3 and αVβ5 integrins and inactivates FAK down stream signaling, leading to suppression of cell proliferation and migration and thus leading to apoptosis (Kamphaus et al. 2000; Panka and Mier, 2003).

α2(IV)NC1 binds to αVβ3 and αVβ5 integrins and initiates two apoptotic pathways that include activation of caspase-8 and -9, (both initiators of the downstream apoptotic process) and leads to activation of caspase-3 (Roth et al. 2005, Magnon et al. 2005). α2(IV)NC1 activates caspase-8 by downregulation of Flip levels. Upregulation of Fas/Fas ligand triggers not only cell death directly through caspase-3 activation but also indirectly through mitochondrial damage via activation of caspase-9 within the apoptosome. On the other hand, phosphorylated FAK/PI3K is known to inactivate the mitochondrial apoptotic pathway by inhibition of caspase-9 (Magnon et al. 2005). So, α2(IV)NC1 directly activates procaspase-9 through inhibition of the FAK/PI3K pathway and amplifies the Fas-dependent pathway in mitochondria. Caspase activation might be exploited for antitumorogenic therapy in the treatment of cancer.

**Figure 2:**
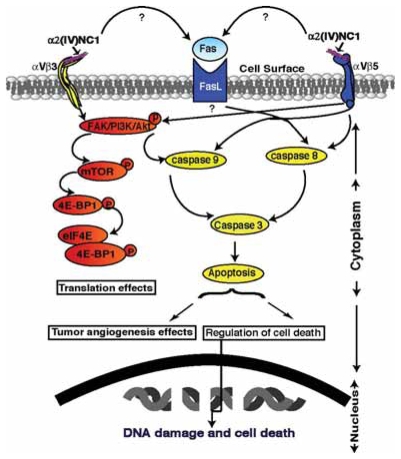
Schematic illustration of distinct signaling pathways induced by α2(IV)NC1. α2(IV)NC1 binds to αVβ3 and αVβ5 integrins on endothelial and tumor cells, and initiates two distinct signaling pathways. (1) Inhibition of phosphorylation of FAK/PI-3K/eIF4E/4E-BP1. (2) Activation of caspase-8 and -9 leading to activation of caspase-3. α2(IV)NC1 activates pro-caspase-8 and -9 directly through inhibition of FAK/PI3K/Akt/mTOR pathway. α2(IV)NC1 also indirectly enhances the mitochondrial pathway through Fas dependent caspase-8 activation, which results in inhibition of protein synthesis, DNA damage and cell death.

Overall α2(IV)NC1 inhibits FAK/Akt signaling by binds to αVβ3 and αVβ5 integrins and induces distinct signaling pathways to activate caspase-3 in endothelial or in tumoral cells. α2(IV)NC1 initiates two apoptotic pathways, involving activation of caspase-8 and -9, leading to activation of caspase-3. (a) α2(IV)NC1 activates procaspase-9 directly through inhibition of the FAK/PI3K/Akt pathway, and (b) activates caspase-3 by amplifying indirectly the mitochondrial pathway through Fas-dependent caspase-8 activation. Where as in tumor cells α2(IV)NC1 activates caspase-3 only the mitochondrial pathway (Magnon et al. 2005) (Fig. 2).

### α3(IV)NC1 or tumstatin

**Figure 3:**
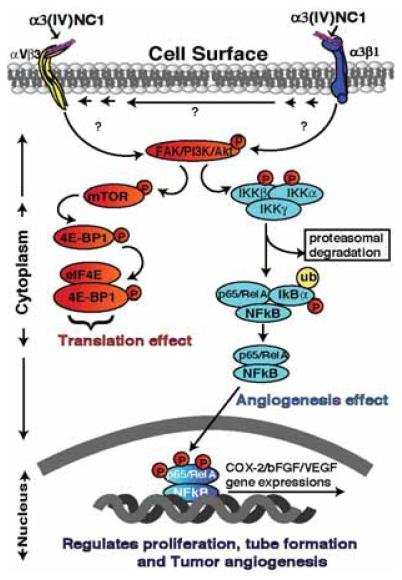
Schematic illustration of different signaling pathway mediated by α3(IV)NC1. α3(IV)NC1 binds to αVβ3 and α3β1 integrins, and inhibits phosphorylation of FAK. Inhibition of FAK activation leads to inhibition of FAK/PI-3K/eIF4E/4E-BP1 cap dependent translation. In addition α3(IV)NC1 inhibits NFκB mediated signaling in hypoxic conditions leading to inhibition of COX-2/VEGF/bFGF expression, resulting in inhibition of hypoxic tumor angiogenesis.

A 28-kDa proteolytic peptide liberated from the NC1 domain of α3 chain of Type IV collagen by MMP-9 and 2, has been shown to inhibit the proliferation of melanoma and other epithelial tumor cell lines *in vitro* by binding to the CD47/αVβ3 integrin complex (Monboisse et al. 1994; Han et al. 1997; Shahan et al. 1999; Petitclerc et al. 2000; Hamano et al. 2003). *In vivo* over expression of α3(IV)NC1 domain in tumor cells inhibited their invasive properties in mouse melanoma model (Pasco et al. 2004; Pasco et al. 2005). α3(IV)NC1 inhibits formation of new blood vessels in Matrigel plugs and suppresses tumor growth of human renal cell carcinoma and prostate carcinoma in mouse xenograft models and this is associated with *in vivo* endothelial cell specific apoptosis (Petitclerc et al. 2000; Maeshima et al. 2000). The antiangiogenic activity of α3(IV)NC1 is localized to two distinct integrin binding region of the molecule that is separate from the region responsible for the antitumor cell activity (Maeshima et al. 2000; Borza et al. 2006; Boosani et al. 2007). αVβ3 binds in the NH2-terminal end (54–132 amino acid region) of the α3(IV)NC1 that is associated with the antiangiogenic activity and α3β1 binds in the COOH-terminal end (185–203 amino acid region) that is associated with the antitumor activity (Shahan et al. 1999; Floquet et al. 2004). These two distinct integrin binding sites of α3(IV)NC1 mediating two distinct antiangiogenic and antitumorogenic activities was recently reported by Boosani et al. (Fig. 3) (Boosani et al. 2007).

The signaling mechanism involving inhibition of endothelial cell-specific protein synthesis by α3(IV)NC1 binding to αVβ3 integrin was reported previously (Maeshima et al. 2002; Sudhakar et al. 2003). This mechanism has since been implicated in inhibition of tumor growth from several tumor cell lines such as CT26 (colon adenocarcinoma), LLC (Lewis lung carcinoma), renal cell carcinoma (786-O), prostate carcinoma (PC3), human prostate cancer (DU145), human lung cancer (H1299), and human fibrosarcoma (HT1080), by inhibiting tumor angiogenesis (Petitclerc et al. 2000; Miyoshi et al. 2006; Borza et al. 2006; Maeshima et al. 2000). The antiangiogenic activity of α3(IV)NC1 upon its interaction with αVβ3 integrin, inhibit activation of FAK, PI3K, Akt/protein kinase B, mTOR pathways and prevents the dissociation of eIF4E protein from 4E-BP1 leading to the inhibition of Cap-dependent translation (Maeshima et al. 2002; Sudhakar et al. 2003). Furthermore, these findings indicate the role for integrins in mediating cell specific inhibition of protein translation that suggests a potential mechanism for the specific effects of α3(IV)NC1 on endothelial cells (Sudhakar et al. 2003).

Recently our laboratory has identified the signaling mechanism mediated by α3(IV)NC1 that inhibits hypoxia induced cyclo-oxygenase-2 (COX-2) expression in endothelial cells via FAK/Akt/NFκB pathways, and leads to decreased tumor angiogenesis and tumor growth in an α3β1 integrin dependent manner (Boosani et al. 2007). COX-2 is a key enzyme involved in conversion of arachidonic acid to prostaglandins (PGs) and other eicosanoids (Hla and Neilson, 1992). Two isoforms of COX were identified; COX-1 is expressed constitutively, whereas COX-2 is induced by a variety of factors, including cytokines, growth factors, and tumor promoters (Hla and Neilson, 1992; DuBois et al. 1994). Mitogens such as tumor necrosis factor, phorbol ester, lipopolysaccharide, or interleukin-1 are known to increase the steady-state levels of COX-2 (Jones et al. 1993; Michiels et al. 1993). Hypoxia induces COX-2 expression by nuclear transcription factor-kappa B (NFκB) (Schmedtje et al. 1997; Tamura et al. 2002). There is ample evidence that COX-2 over expression contributes to carcinogenesis and that COX-2 disruption can both prevent and treat a variety of solid tumors (Wu et al. 2003; Wu et al. 2004; Tamura et al. 2002; Subbaramaiah et al. 1997). NFκB plays an essential role in many diseases such as AIDS, atherosclerosis, asthma, arthritis, diabetes, inflammatory bowel disease, muscular dystrophy, stroke, viral infections, cancer and is a possible target of therapeutic intervention (Kumar et al. 2004; Shishodia and Aggarwal, 2004). NFκB may facilitate the induction of COX-2 by lipopolysaccharide and phorbol ester in concert with the nuclear factor-interleukin-6 expression site and a cAMP responsive element site in bovine aortic endothelial cells (Inoue et al. 1995; Yamamoto et al. 1995).

In endothelial cells, α3(IV)NC1 binds to α3β1 integrins and inhibits NFκB signaling resulting in inhibition of COX-2 mediated signaling. It was further proved that expression of COX-2 was inhibited in β3 integrin knockout endothelial cells upon treatment with α3(IV)NC1, indicating that COX-2 mediated signaling is regulated through α3β1 and not by αVβ3 integrin (Boosani et al. 2007). Interestingly COX-2 expression was not affected when hypoxic α3 integrin knockout ECs were treated with α3(IV)NC1 protein, confirming that COX-2 expression was regulated by α3β1 integrin (Boosani et al. 2007). These findings strongly suggest that α3(IV)NC1 has the ability to inhibit pro-inflammatory factor COX-2, and inhibit tumor vasculature and tumor growth in an α3β1 integrin dependent manner (Boosani et al. 2007). In addition to COX-2 inhibition, the COX-2 regulated down stream VEGF and bFGF protein expression was also inhibited upon α3(IV)NC1 treatment to endothelial cells (Boosani et al. 2007). COX-2 was also reported to play a key role in tumor angiogenesis (Leung et al. 2003; Harris, 2002). Moreover, several investigators have demonstrated that blockade of the COX-2 mediated pathway serves as a therapeutic benefit in different cancer models (Gately and Kerbel, 2003; Panka and Mier, 2003; Kunz and Ibrahim, 2003). COX-2 regulates cellular responses to pathological conditions and studies have demonstrated that COX-2 is a potential target for tumor angiogenesis (Kunz and Ibrahim, 2003; Gately and Kerbel, 2003; Kunz et al. 2003).

The antitumorogenic activity of α3(IV)NC1 under hypoxic conditions in solid tumors was not clearly understood earlier. Our studies shed light on this mechanism by demonstrating that α3(IV)NC1 binds to α3β1 integrins which inhibit COX-2 expression both *in vitro* and *in vivo* (Boosani et al. 2007). It is clear that inhibition of hypoxia induced angiogenesis by α3(IV)NC1 is a complex process requiring further investigation. Our previous findings indicate that there may be several targets for the inhibitory effects of α3(IV)NC1 on tumor-angiogenesis, including or in addition to COX-2, VEGF and bFGF (Boosani et al. 2007).

In summary, the *in vitro* and *in vivo* observations support the role of αVβ3 and α3β1 integrins for the antiangiogenic activity of α3(IV)NC1. While both these integrins mediate tube formation in cultured ECs, α3β1 integrin mediates signaling events that influences downstream effects of COX-2 expression which appears to be central to the mechanism of α3(IV)NC1 antitumor activities. Our studies also demonstrate that α3(IV)NC1 inhibits hypoxia induced angiogenesis by (1) inhibiting NFκB activation, leading to (2) inhibition of COX-2 expression, which in turn results in (3) down regulation of hypoxia induced VEGF/bFGF expression (Fig. 3) (Boosani et al. 2007). These findings have potential implications of α3(IV)NC1 for treatment of solid tumor growth, which depend critically on hypoxic angiogenesis. The decrease in COX-2 expression under hypoxia that results in decreased VEGF/bFGF expression will likely represent a primary molecular mechanism by which α3(IV)NC1 inhibit the pathological angiogenesis that is essential to the growth of tumors (Boosani et al. 2007).

### α6(IV)NC1

In addition to the NC1 domains of collagen IV α1,α2, α3 chains, α6(IV)NC1 domain also possesses antiangiogenic activity and inhibits tumor growth (Petitclerc et al. 2000), but a clear and extensive analysis of this molecule are yet to be unraveled.

## Conclusions and Future Directions

Type IV collagen derived endogenous angiogenesis inhibitors bind to different cell surface integrins and exert their effects through multiple mechanisms that include induction of endothelial cells apoptosis, inhibition of migration, proliferation, tube formation of endothelial cells, and inhibit or alter the functions of proangiogenic growth factors. Three possible conclusions can be drawn from the signaling mechanisms of Type IV collagen derived angiogenic inhibitors that are shown in Table 1. (1) All these collagen type IV derived inhibitors appears to exert their antiangiogenic effects by binding to specific cell surface integrins. (2) These inhibitors also block the binding of natural ligand/binding partners for proangiogenic receptors/molecules. (3) In addition, possibly by binding to its receptors, these inhibitors crosstalk with other cell surface receptors and activate specific caspase mediated signaling to regulate cell function (Panka and Mier, 2003; Magnon et al. 2005).

Currently, more than 25 different endogenous circulating molecules (small proteins or peptides) are found to exist in the human body that functions as angiogenesis inhibitors. Circulating physiological concentration of α3(IV)NC1 was reported in normal mice to be about 336 ng/ml, that was absent in α3 chain of Type IV Collagen null mice (Hamano et al. 2003). Administration of 300 ng of recombinant α3(IV)NC1 to physiological levels in α3 chain of Type IV Collagen null mice with LLC tumors showed decrease tumor growth, the number of blood vessels and circulating endothelial cells to the wild-type baseline levels (Hamano et al. 2003; Sund et al. 2005). It is quite possible that genetic control of the physiologic levels of these endogenous angiogenesis inhibitors might contribute to a critical line of defense against the conversion of dormant neoplastic events into a malignant phenotype of cancer.

Several angiogenic inhibitors including integrin αV antagonist EMD 121974, 2-methoxyestradiol (panzam) and, MMP-2 and -9 inhibitor COL-3 etc are currently in phase 1/2 human clinical trails (Jansen et al. 2004). Questions regarding resistance to these angiogenesis inhibitors do remain unanswered; however, a combination of radiation therapy with other antiangiogenic therapies may also prove to be clinically useful and effective. Further evaluation through extensive laboratory studies on these molecules are needed to address the function of Type IV collagen derived endogenous inhibitors of angiogenesis to be considered for the clinical trials. Earlier lessons from preclinical trials of angiostatin, endostatin, Thrombospondin-1 (ABT-510) and 2-ME suggest that more basic laboratory research studies are required to better understand the mechanism of actions associated with each of these endogenous angiogenesis inhibitor molecules. Presently, some of the anti-angiogenic agents such as Bevacizumab and several other VEGFR tyrosine kinase inhibitors; Vatalanib (PTK787/ZK 222584), Semaxanib (SU5416), Sunitinib (SU11248), Sorafenib (BAY 43-9006) are in clinical trials (Hurwitz et al. 2004; Morabito et al. 2006). In the past few years several advances were made VBM derived endogenous angiogenesis inhibitors functional studies. VBM not only is an important structural component of the blood capillary, but it is also an important functional regulator of tumor angiogenesis and tumor growth. VBM in an assembled form performs completely new role compared with degraded form (exposed to different proteases). The degraded VBM modulate cellular behavior, hiding or exposing basement membrane integrin binding sequences. Therefore, VBM has become very good source of a collection of peptides or proteins that posses distinct activities with in the same primary sequence. These sequences are available at different stages during VBM structural changes; just like as the coagulation pathway proteins. Our understanding of how these collagen Type IV derived angiogenesis inhibitors regulate angiogenesis has just began compared to type XVIII collagen derived angiogenesis inhibitor or endostatin. Further extensive laboratory studies are required to know how Type IV collagen derived molecules regulating cellular functions to halt tumor growth and tumor angiogenesis.

## Figures and Tables

**Table 1:** Signaling mechanisms mediated by type IV collage derived angiogenesis inhibitors.

Angiogenesis inhibitor name	Human α1(IV)NC1	Human α2(IV)NC1	Human α3(IV)NC1
Inhibitor origin	α1 Type IV collagen	α2 Type IV collagen	α3 Type IV collagen
Generation of inhibitor	By MMP-9 and -2	By MMP-9 and -2	By MMP-9 and -2
Receptors	α1β1 integrin	αVβ5/αVβ3 integrins	αVβ3/α3β1 integrins
Proliferation	Inhibition	Inhibition	Inhibition
Migration	Inhibition	Inhibition	No effect
Tube formation	Inhibition	Inhibition	Inhibition
Mechanism of action	FAK, Ras, c-Raf, MEK1/2, p38, ERK1/2, HIF1α mediated signaling	FAK, Akt, PI3K/mTOR/eIF-4E/4E-BP1 signaling and FasL mediated apoptosis	FAK, Akt, PI3K/mTOR/eIF-4E/4E-BP1 and NFkB/COX-2 mediated signaling

## Keywords

VBM: vascular basement membrane
ECM: extra cellular matrix
MMP: matrix metalloproteinase
HUVEC: human umbilical vein endothelial cell
MLEC: mouse lung endothelial cells
SCC-PSA1: teratocarcinoma cell line
VEGF: vascular endothelial cell growth factor
bFGF: basic fibroblast growth factor
α1–α6(IV)NC1: non-collagenous α1–α6 chains of Type IV collagen domains

## References

[b1-grsb-2007-217] Albig AR Schiemann WP 2004 DNA Cell Biol 23 367 79 15231070 10.1089/104454904323145254

[b2-grsb-2007-217] Aumailley M Timpl R 1986 J Cell Biol 103 1569 75 3771647 10.1083/jcb.103.4.1569 PMC2114329

[b3-grsb-2007-217] Boosani CS Mannam AP Cosgrove D Silva R Hodivala-Dilke KM Keshamouni VG Sudhakar A 2007 Blood 110 1168 77 17426256 10.1182/blood-2007-01-066282 PMC1939900

[b4-grsb-2007-217] Boosani CS Sudhakar A 2006 Protein Expr Purif 49 211 8 16631378 10.1016/j.pep.2006.03.007

[b5-grsb-2007-217] Borza CM Pozzi A Borza DB Pedchenko V Hellmark T Hudson BG Zent R 2006 J Biol Chem 10.1074/jbc.M601147200 16731529

[b6-grsb-2007-217] Brew K Dinakarpandian D Nagase H 2000 Biochim Biophys Acta 1477 267 83 10708863 10.1016/s0167-4838(99)00279-4

[b7-grsb-2007-217] Brooks PC Silletti S von Schalscha TL Friedlander M Cheresh DA 1998 Cell 92 391 400 9476898 10.1016/s0092-8674(00)80931-9

[b8-grsb-2007-217] Carmeliet P Dor Y Herbert JM Fukumura D Brusselmans K Dewerchin M Neeman M Bono F Abramovitch R Maxwell P Koch CJ Ratcliffe P Moons L Jain RK Collen D Keshert E Keshet E 1998 Nature 394 485 90 9697772 10.1038/28867

[b9-grsb-2007-217] Chelberg MK McCarthy JB Skubitz AP Furcht LT Tsilibary EC 1990 J Cell Biol 111 261 70 2365734 10.1083/jcb.111.1.261 PMC2116148

[b10-grsb-2007-217] Colorado PC Torre A Kamphaus G Maeshima Y Hopfer H Takahashi K Volk R Zamborsky ED Herman S Sarkar PK Ericksen MB Dhanabal M Simons M Post M Kufe DW Weichselbaum RR Sukhatme VP Kalluri R 2000 Cancer Res 60 2520 6 10811134

[b11-grsb-2007-217] Cornelius LA Nehring LC Harding E Bolanowski M Welgus HG Kobayashi DK Pierce RA Shapiro SD 1998 J Immunol 161 6845 52 9862716

[b12-grsb-2007-217] Dinney CP Bielenberg DR Perrotte P Reich R Eve BY Bucana CD Fidler IJ 1998 Cancer Res 58 808 14 9485039

[b13-grsb-2007-217] DuBois RN Tsujii M Bishop P Awad JA Makita K Lanahan A 1994 Am J Physiol 266 G822 7 8203528 10.1152/ajpgi.1994.266.5.G822

[b14-grsb-2007-217] Ferrara N Clapp C Weiner R 1991 Endocrinology 129 896 900 1855480 10.1210/endo-129-2-896

[b15-grsb-2007-217] Floquet N Pasco S Ramont L Derreumaux P Laronze JY Nuzillard JM Maquart FX Alix AJ Monboisse JC 2004 J Biol Chem 279 2091 100 14583633 10.1074/jbc.M307736200

[b16-grsb-2007-217] Folkman J 1971 N Engl J Med 285 1182 6 4938153 10.1056/NEJM197111182852108

[b17-grsb-2007-217] Folkman J 1995a Nat Med 1 27 31 7584949 10.1038/nm0195-27

[b18-grsb-2007-217] Folkman J 1995b Mol Med 1 120 2 8529090 PMC2229937

[b19-grsb-2007-217] Folkman J 2003 Semin Cancer Biol 13 159 67 12654259 10.1016/s1044-579x(02)00133-5

[b20-grsb-2007-217] Gately S Kerbel R 2003 Prog Exp Tumor Res 37 179 92 12795055 10.1159/000071373

[b21-grsb-2007-217] Good DJ Polverini PJ Rastinejad F Le Beau MM Lemons RS Frazier WA Bouck NP 1990 Proc Natl Acad Sci USA 87 6624 8 1697685 10.1073/pnas.87.17.6624 PMC54589

[b22-grsb-2007-217] Hamano Y Zeisberg M Sugimoto H Lively JC Maeshima Y Yang C Hynes RO Werb Z Sudhakar A Kalluri R 2003 Cancer Cell 3 589 601 12842087 10.1016/s1535-6108(03)00133-8 PMC2775452

[b23-grsb-2007-217] Han J Ohno N Pasco S Monboisse JC Borel JP Kefalides NA 1997 J Biol Chem 272 20395 401 9252346 10.1074/jbc.272.33.20395

[b24-grsb-2007-217] Hanahan D Folkman J 1996 Cell 86 353 64 8756718 10.1016/s0092-8674(00)80108-7

[b25-grsb-2007-217] Harris AL 2002 Nat Rev Cancer 2 38 47 11902584 10.1038/nrc704

[b26-grsb-2007-217] Hla T Neilson K 1992 Proc Natl Acad Sci USA 89 7384 8 1380156 10.1073/pnas.89.16.7384 PMC49714

[b27-grsb-2007-217] Hudson BG Kalluri R Gunwar S Noelken ME 1994 Contrib Nephrol 107 163 7 8004963 10.1159/000422975

[b28-grsb-2007-217] Hudson BG Reeders ST Tryggvason K 1993 J Biol Chem 268 26033 6 8253711

[b29-grsb-2007-217] Hudson BG Tryggvason K Sundaramoorthy M Neilson EG 2003 N Engl J Med 348 2543 56 12815141 10.1056/NEJMra022296

[b30-grsb-2007-217] Hurwitz H Fehrenbacher L Novotny W Cartwright T Hainsworth J Heim W Berlin J Baron A Griffing S Holmgren E Ferrara N Fyfe G Rogers B Ross R Kabbinavar F 2004 N Engl J Med 350 2335 42 15175435 10.1056/NEJMoa032691

[b31-grsb-2007-217] Hynes RO 2002 Nat Med 8 918 21 12205444 10.1038/nm0902-918

[b32-grsb-2007-217] Inoue H Yokoyama C Hara S Tone Y Tanabe T 1995 J Biol Chem 270 24965 71 7559624 10.1074/jbc.270.42.24965

[b33-grsb-2007-217] Jansen M de Witt Hamer PC Witmer AN Troost D van Noorden CJ 2004 Brain Res Brain Res Rev 45 143 63 15210301 10.1016/j.brainresrev.2004.03.001

[b34-grsb-2007-217] Jiang Y Goldberg ID Shi YE 2002 Oncogene 21 2245 52 11948407 10.1038/sj.onc.1205291

[b35-grsb-2007-217] Jones DA Carlton DP McIntyre TM Zimmerman GA Prescott SM 1993 J Biol Chem 268 9049 54 8473346

[b36-grsb-2007-217] Kamphaus GD Colorado PC Panka DJ Hopfer H Ramchandran R Torre A Maeshima Y Mier JW Sukhatme VP Kalluri R 2000 J Biol Chem 275 1209 1215 10625665 10.1074/jbc.275.2.1209

[b37-grsb-2007-217] Keely PJ Wu JE Santoro SA 1995 Differentiation 59 1 13 7589890 10.1046/j.1432-0436.1995.5910001.x

[b38-grsb-2007-217] Kendall RL Thomas KA 1993 Proc Natl Acad Sci USA 90 10705 9 8248162 10.1073/pnas.90.22.10705 PMC47846

[b39-grsb-2007-217] Kieran MW Folkman J Heymach J 2003 Nat Med 9 1104 author reply 1104–5 12949515 10.1038/nm0903-1104a

[b40-grsb-2007-217] Kim YM Hwang S Pyun BJ Kim TY Lee ST Gho YS Kwon YG 2002 J Biol Chem 277 27872 9 12029087 10.1074/jbc.M202771200

[b41-grsb-2007-217] Kuhn K 1995 Matrix Biol 14 439 45 7795882 10.1016/0945-053x(95)90001-2

[b42-grsb-2007-217] Kuhn K Wiedemann H Timpl R Risteli J Dieringer H Voss T Glanville RW 1981 FEBS Lett 125 123 8 6262125 10.1016/0014-5793(81)81012-5

[b43-grsb-2007-217] Kumar A Takada Y Boriek AM Aggarwal BB 2004 J Mol Med 82 434 48 15175863 10.1007/s00109-004-0555-y

[b44-grsb-2007-217] Kung AL Wang S Klco JM Kaelin WG Livingston DM 2000 Nat Med 6 1335 40 11100117 10.1038/82146

[b45-grsb-2007-217] Kunz M Ibrahim SM 2003 Mol Cancer 2 23 12740039 10.1186/1476-4598-2-23 PMC155638

[b46-grsb-2007-217] Kunz M Moeller S Koczan D Lorenz P Wenger RH Glocker MO Thiesen HJ Gross G Ibrahim SM 2003 J Biol Chem 278 45651 60 12939282 10.1074/jbc.M301373200

[b47-grsb-2007-217] Kusafuka K Hiraki Y Shukunami C Kayano T Takemura T 2002 Acta Histochem 104 167 75 12086337 10.1078/0065-1281-00642

[b48-grsb-2007-217] Lee JW Bae SH Jeong JW Kim SH Kim KW 2004 Exp Mol Med 36 1 12 15031665 10.1038/emm.2004.1

[b49-grsb-2007-217] Lee TH Rhim T Kim SS 1998 J Biol Chem 273 28805 12 9786880 10.1074/jbc.273.44.28805

[b50-grsb-2007-217] Leung WK To KF Go MY Chan KK Chan FK Ng EK Chung SC Sung JJ 2003 Int J Oncol 23 1317 22 14532971

[b51-grsb-2007-217] Lingen MW Polverini PJ Bouck NP 1998 Cancer Res 58 5551 8 9850093

[b52-grsb-2007-217] Mabjeesh NJ Escuin D LaVallee TM Pribluda VS Swartz GM Johnson MS Willard MT Zhong H Simons JW Giannakakou P 2003 Cancer Cell 3 363 75 12726862 10.1016/s1535-6108(03)00077-1

[b53-grsb-2007-217] Madri JA 1997 Transpl Immunol 5 179 83 9402683 10.1016/s0966-3274(97)80035-4

[b54-grsb-2007-217] Maeshima Y Colorado PC Torre A Holthaus KA Grunkemeyer JA Ericksen MB Hopfer H Xiao Y Stillman IE Kalluri R 2000 J Biol Chem 275 21340 8 10766752 10.1074/jbc.M001956200

[b55-grsb-2007-217] Maeshima Y Sudhakar A Lively JC Ueki K Kharbanda S Kahn CR Sonenberg N Hynes RO Kalluri R 2002 Science 295 140 3 11778052 10.1126/science.1065298

[b56-grsb-2007-217] Magnon C Galaup A Mullan B Rouffiac V Bouquet C Bidart JM Griscelli F Opolon P Perricaudet M 2005 Cancer Res 65 4353 61 15899827 10.1158/0008-5472.CAN-04-3536

[b57-grsb-2007-217] Magnon C Opolon P Ricard M Connault E Ardouin P Galaup A Metivier D Bidart JM Germain S Perricaudet M Schlumberger M 2007 J Clin Invest 117 1844 1855 17557121 10.1172/JCI30269 PMC1884687

[b58-grsb-2007-217] Maione TE Gray GS Petro J Hunt AJ Donner AL Bauer SI Carson HF Sharpe RJ 1990 Science 247 77 9 1688470 10.1126/science.1688470

[b59-grsb-2007-217] Marneros AG Olsen BR 2001 Matrix Biol 20 337 45 11566268 10.1016/s0945-053x(01)00151-2

[b60-grsb-2007-217] Michiels C Arnould T Knott I Dieu M Remacle J 1993 Am J Physiol 264 C866 74 8476019 10.1152/ajpcell.1993.264.4.C866

[b61-grsb-2007-217] Miller JW Adamis AP Shima DT D’Amore PA Moulton RS O’Reilly MS Folkman J Dvorak HF Brown LF Berse B 1994 Am J Pathol 145 574 84 7521577 PMC1890317

[b62-grsb-2007-217] Miyoshi T Hirohata S Ogawa H Doi M Obika M Yonezawa T Sado Y Kusachi S Kyo S Kondo S Shiratori Y Hudson BG Ninomiya Y 2006 Faseb J 20 1904 6 16877525 10.1096/fj.05-5565fje

[b63-grsb-2007-217] Monboisse JC Garnotel R Bellon G Ohno N Perreau C Borel JP Kefalides NA 1994 J Biol Chem 269 25475 82 7929248

[b64-grsb-2007-217] Morabito A De Maio E Di Maio M Normanno N Perrone F 2006 Oncologist 11 753 64 16880234 10.1634/theoncologist.11-7-753

[b65-grsb-2007-217] Moses MA Wiederschain D Wu I Fernandez CA Ghazizadeh V Lane WS Flynn E Sytkowski A Tao T Langer R 1999 Proc Natl Acad Sci USA 96 2645 50 10077564 10.1073/pnas.96.6.2645 PMC15822

[b66-grsb-2007-217] O’Reilly MS Boehm T Shing Y Fukai N Vasios G Lane WS Flynn E Birkhead JR Olsen BR Folkman J 1997 Cell 88 277 85 9008168 10.1016/s0092-8674(00)81848-6

[b67-grsb-2007-217] O’Reilly MS Pirie-Shepherd S Lane WS Folkman J 1999 Science 285 1926 8 10489375 10.1126/science.285.5435.1926

[b68-grsb-2007-217] Panka DJ Mier JW 2003 J Biol Chem 278 37632 6 12876280 10.1074/jbc.M307339200

[b69-grsb-2007-217] Pasco S Brassart B Ramont L Maquart FX Monboisse JC 2005 Cancer Detect Prev 29 260 6 15936594 10.1016/j.cdp.2004.09.003

[b70-grsb-2007-217] Pasco S Monboisse JC Kieffer N 2000 J Biol Chem 275 32999 3007 10934203 10.1074/jbc.M005235200

[b71-grsb-2007-217] Pasco S Ramont L Venteo L Pluot M Maquart FX Monboisse JC 2004 Exp Cell Res 301 251 65 15530861 10.1016/j.yexcr.2004.07.036

[b72-grsb-2007-217] Patterson BC Sang QA 1997 J Biol Chem 272 28823 5 9360944 10.1074/jbc.272.46.28823

[b73-grsb-2007-217] Petitclerc E Boutaud A Prestayko A Xu J Sado Y Ninomiya Y Sarras MP Jr Hudson BG Brooks PC 2000 J Biol Chem 275 8051 61 10713126 10.1074/jbc.275.11.8051

[b74-grsb-2007-217] Pike SE Yao L Jones KD Cherney B Appella E Sakaguchi K Nakhasi H Teruya-Feldstein J Wirth P Gupta G Tosato G 1998 J Exp Med 188 2349 56 9858521 10.1084/jem.188.12.2349 PMC2212424

[b75-grsb-2007-217] Pike SE Yao L Setsuda J Jones KD Cherney B Appella E Sakaguchi K Nakhasi H Atreya CD Teruya-Feldstein J Wirth P Gupta G Tosato G 1999 Blood 94 2461 8 10498619

[b76-grsb-2007-217] Pozzi A Moberg PE Miles LA Wagner S Soloway P Gardner HA 2000 Proc Natl Acad Sci USA 97 2202 7 10681423 10.1073/pnas.040378497 PMC15778

[b77-grsb-2007-217] Ries A Engel J Lustig A Kuhn K 1995 J Biol Chem 270 23790 4 7559554 10.1074/jbc.270.40.23790

[b78-grsb-2007-217] Roth JM Akalu A Zelmanovich A Policarpio D Ng B MacDonald S Formenti S Liebes L Brooks PC 2005 Am J Pathol 166 901 11 15743801 10.1016/s0002-9440(10)62310-3 PMC1602358

[b79-grsb-2007-217] Schmedtje JF Jr Ji YS Liu WL DuBois RN Runge MS 1997 J Biol Chem 272 601 8 8995303 10.1074/jbc.272.1.601

[b80-grsb-2007-217] Semenza GL 2003 Nat Rev Cancer 3 721 32 13130303 10.1038/nrc1187

[b81-grsb-2007-217] Senger DR Perruzzi CA Streit M Koteliansky VE de Fougerolles AR Detmar M 2002 Am J Pathol 160 195 204 11786413 10.1016/s0002-9440(10)64363-5 PMC1867136

[b82-grsb-2007-217] Shahan TA Ziaie Z Pasco S Fawzi A Bellon G Monboisse JC Kefalides NA 1999 Cancer Res 59 4584 90 10493512

[b83-grsb-2007-217] Shiojima I Walsh K 2002 Circ Res 90 1243 50 12089061 10.1161/01.res.0000022200.71892.9f

[b84-grsb-2007-217] Shishodia S Aggarwal BB 2004 Biochem Pharmacol 68 1071 80 15313403 10.1016/j.bcp.2004.04.026

[b85-grsb-2007-217] Strieter RM Polverini PJ Arenberg DA Kunkel SL 1995a Shock 4 155 60 8574748 10.1097/00024382-199509000-00001

[b86-grsb-2007-217] Strieter RM Polverini PJ Kunkel SL Arenberg DA Burdick MD Kasper J Dzuiba J Van Damme J Walz A Marriott D 1995b J Biol Chem 270 27348 57 7592998 10.1074/jbc.270.45.27348

[b87-grsb-2007-217] Subbaramaiah K Zakim D Weksler BB Dannenberg AJ 1997 Proc Soc Exp Biol Med 216 201 10 9349689 10.3181/00379727-216-44170

[b88-grsb-2007-217] Sudhakar A Nyberg P Keshamouni VG Mannam AP Li J Sugimoto H Cosgrove D Kalluri R 2005 J Clin Invest 115 2801 10 16151532 10.1172/JCI24813 PMC1199529

[b89-grsb-2007-217] Sudhakar A Sugimoto H Yang C Lively J Zeisberg M Kalluri R 2003 Proc Natl Acad Sci USA 100 4766 71 12682293 10.1073/pnas.0730882100 PMC153630

[b90-grsb-2007-217] Sund M Hamano Y Sugimoto H Sudhakar A Soubasakos M Yerramalla U Benjamin LE Lawler J Kieran M Shah A Kalluri R 2005 Proc Natl Acad Sci USA 102 2934 9 15710885 10.1073/pnas.0500180102 PMC549486

[b91-grsb-2007-217] Tamura M Sebastian S Gurates B Yang S Fang Z Bulun SE 2002 J Clin Endocrinol Metab 87 3504 7 12107271 10.1210/jcem.87.7.8796

[b92-grsb-2007-217] Timpl R 1996 Curr Opin Cell Biol 8 618 24 8939648 10.1016/s0955-0674(96)80102-5

[b93-grsb-2007-217] Timpl R Wiedemann H van Delden V Furthmayr H Kuhn K 1981 Eur J Biochem 120 203 11 6274634 10.1111/j.1432-1033.1981.tb05690.x

[b94-grsb-2007-217] Tsilibary EC Reger LA Vogel AM Koliakos GG Anderson SS Charonis AS Alegre JN Furcht LT 1990 J Cell Biol 111 1583 91 2211826 10.1083/jcb.111.4.1583 PMC2116235

[b95-grsb-2007-217] Unruh A Ressel A Mohamed HG Johnson RS Nadrowitz R Richter E Katschinski DM Wenger RH 2003 Oncogene 22 3213 20 12761491 10.1038/sj.onc.1206385

[b96-grsb-2007-217] Vandenberg P Kern A Ries A Luckenbill-Edds L Mann K Kuhn K 1991 J Cell Biol 113 1475 83 1646206 10.1083/jcb.113.6.1475 PMC2289033

[b97-grsb-2007-217] Volpert OV Zaichuk T Zhou W Reiher F Ferguson TA Stuart PM Amin M Bouck NP 2002 Nat Med 8 349 57 11927940 10.1038/nm0402-349

[b98-grsb-2007-217] Wu AW Gu J Li ZF Ji JF Xu GW 2004 World J Gastroenterol 10 2323 6 15285012 10.3748/wjg.v10.i16.2323 PMC4576281

[b99-grsb-2007-217] Wu G Mannam AP Wu J Kirbis S Shie JL Chen C Laham RJ Sellke FW Li J 2003 Am J Physiol Heart Circ Physiol 285 H2420 9 12881220 10.1152/ajpheart.00187.2003

[b100-grsb-2007-217] Yamamoto K Arakawa T Ueda N Yamamoto S 1995 J Biol Chem 270 31315 20 8537402 10.1074/jbc.270.52.31315

[b101-grsb-2007-217] Yi M Ruoslahti E 2001 Proc Natl Acad Sci USA 98 620 4 11209058 10.1073/pnas.98.2.620 PMC14637

[b102-grsb-2007-217] Yurchenco PD O’Rear JJ 1994 Curr Opin Cell Biol 6 674 81 7833047 10.1016/0955-0674(94)90093-0

[b103-grsb-2007-217] Zutter MM Santoro SA 1990 Am J Pathol 137 113 20 2164774 PMC1877693

